# Epidemiology and Treatment of Metastatic Lesions Around the Elbow: A Systematic Review

**DOI:** 10.3390/jcm14176297

**Published:** 2025-09-06

**Authors:** Andrea De Fazio, Giovan Giuseppe Mazzella, Guglielmo Miele, Maria Beatrice Bocchi, Omar El Ezzo, Giacomo Capece, Giulio Maccauro, Raffaele Vitiello

**Affiliations:** 1 Department of Orthopaedics, Fondazione Policlinico Universitario Agostino Gemelli IRCCS, 00168 Rome, Italy; andrea.defazio01@icatt.it (A.D.F.); giovangiuseppe.mazzella01@icatt.it (G.G.M.); m.beabocchi@gmail.com (M.B.B.); omar.elezzo@guest.policlinicogemelli.it (O.E.E.); giacomo.capece01@icatt.it (G.C.); giulio.maccauro@policlinicogemelli.it (G.M.); lele.vitiello@gmail.com (R.V.); 2 Orthopedics and Traumatology, Catholic University of the Sacred Heart, 00168 Rome, Italy

**Keywords:** elbow, metastases, custom-made, osteolytic lesion, elbow replacement, megaprosthesis, bone resection, oncology

## Abstract

**Introduction**: The elbow is a rare site for bone tumors, and for this reason, the literature provides little data on the epidemiology of metastatic lesions involving the distal humerus, proximal ulna, and radius. Before performing surgery of the metastatic bone, it is first necessary to consider both patients’ and metastatic lesions’ features in order to better choose the best possible treatment. This systematic review aims to collect data on elbow metastases, delineate primary tumors leading to such metastases, guide surgical treatment decisions, and evaluate reconstructive techniques and associated complications. **Material and Methods**: A systematic literature review was conducted in April 2024, searching the PubMed, MEDLINE, and Cochrane Library databases using specific search terms related to elbow metastases. The Preferred Reporting Items for Systematic Reviews and Meta-analyses (PRISMA) was followed. Eligible studies reported at least one patient with metastatic bone disease involving the elbow region and specified the undertaken treatment. For studies reporting multiple skeletal sites, only elbow-specific data were extracted. We excluded recurrences of primary elbow tumors. The methodological quality of included studies was assessed with the modified Coleman Methodology Score (mCMS). **Results**: In total, 28 articles (103 patients) were included. The studies were predominantly case reports (68%), with a mean mCMS of 31. Gender was reported for only 41 patients: 71% were male and 29% female. The mean age at diagnosis of elbow metastatic lesion was 55 years old. Renal cell carcinoma was the most common primary tumor (28%), followed by breast (9%) and lung cancer (6%). The distal humerus was the most frequently affected site (85%). A surgical approach was adopted in 90% of cases, whereas 10% of patients were managed conservatively. Forty-five patients underwent wide tumor resection followed by reconstructive surgery while forty-eight patients received a surgical treatment for either pathological fractures or impending fractures. **Conclusions**: When treating elbow metastasis, a thorough evaluation of the patient is crucial, considering the patient’s functional status, pain management needs, and overall prognosis; all these features influence the treatment of choice. The selected treatment should aim to provide optimal functional outcomes and minimize complications. For patients with pathological or impending fractures, single or double plate fixation is typically the preferred approach. For patients with severe, symptomatic lesions unresponsive to conservative therapy, resection followed by the implantation of a modular prosthesis usually offers the best clinical and functional outcomes.

## 1. Introduction

The elbow is a rare site for bone tumors, which can either be primary, such as sarcomas [1,2], or secondary metastases of other tumors. The literature provides little data on the epidemiology of metastatic lesions involving the distal humerus, proximal ulna, and radius. A. de Geyer et al. reported that humeral metastases are usually secondary to primary breast (17–31%), kidney (13–15%), or lung (11–24%) cancer and that they occur in the midshaft in 42% to 61% of cases and in the proximal humerus in 32% to 45% of cases. However, A. de Geyer et al. did not provide any clear data on the incidence of distal humerus metastasis [2]. Henrichs et al., in their study, reported that <1% of metastatic bone lesions involve the distal humerus [3]. There is a lack of reliable evidence on metastatic involvement of the ulna or proximal radius.

Regarding the ulna, data on primary bone tumors are extremely limited: in the Rizzoli archives, ulnar involvement constituted only 0.9% of all primary bone tumors [4], and in a single-center series, only 23 cases were identified over more than 30 years [5].

Because of the lack of information on metastatic lesions of the elbow, there are no precise guidelines to support the surgeon when they face such a condition. Before performing surgery of the metastatic bone, it is first necessary to consider both the patient’s and the metastatic lesion’s features, such as pain, comorbidity, expected life span, the location of the tumor in the metastatic bone, and the histology of the metastatic tumor [6]. In order to provide patients with the best possible treatment of the metastatic lesion, with an early functional assessment, pain control, and evaluation of the impending fracture risk, the orthopedic surgeon should be involved at an early stage before the metastatic lesion causes a pathological fracture [7]. Bone affected by metastatic cancer is weakened, and whenever there is a surgical indication, the stabilization performed or the prosthetic replacement should stabilize the entire bone affected and should last the remainder of the patient’s life [8]. Choosing the best treatment options is crucial since a wrong decision can potentially affect the patient’s quality of life and survival, especially in cases of pathological fracture related to bone metastasis [9]. According to Weber KL et al., palliative radiation or limited surgery are the most common treatment in patients with elbow metastasis [10]. Since achieving disease-free oncological margins around the elbow should always be the surgeon’s main goal, at times, a less invasive surgical approach does not allow for performing radical resections of the metastatic lesions, so it is necessary to perform more invasive resections. Before the 1970s, this goal was usually achieved by conducting limb amputations, and the occurrence of a pathological fracture through skeletal metastasis was an absolute contraindication for limb salvage surgery. As the population ages, the number and frequency of metastatic lesions are increasing, so orthopedic surgery aims to preserve limbs and functions as much as possible [11]. When wide resection is necessary to obtain disease-free margins, it can be followed by reconstructive surgery. According to the criteria of Capanna and Campanacci, resection with arthroplasty reconstruction should be performed in case of pathological fractures or in case of lesions with a high risk of fracture if the patient’s survival is expected to be more than 6–12 months [12].

Katagiri et al. proposed a prognostic scoring system for patients with skeletal metastases based on clinical and oncological variables, which has been shown to predict survival more accurately than previous models and may help guide treatment selection [13].

Of the long bones, the humerus is the second most affected by pathological fracture after the femur, so reconstructive surgery plays an important role in case of a metastatic lesion of the elbow [14]. The most common reconstructive techniques are arthrodesis, osteoarticular allograft, allograft–prosthesis composite (APC), and prosthetic reconstruction [15]. Elbow reconstruction using contemporary surgical techniques can provide patients with the necessary function to be self-sufficient in the routine activities of daily living, though reconstructive surgery may not be an option if the metastatic lesion affects vital neurovascular structures, which could lead to loss of function of the elbow, wrist, and hand after resection in spite of the reconstructive technique adopted [10]. The scientific literature does not provide a unanimous answer on which reconstructive technique has better outcomes and fewer complications: on the one hand, reconstruction using an allograft can lead to elbow instability, pain, high rates of major complications, and poor functional outcomes [15]; total elbow prosthesis, on the other hand, is associated with complications such as loosening, revision, and infections [16]. Caredda M. et al. showed good functional outcomes in elderly patients with distal humerus fractures and poor bone stock undergoing elbow reconstruction using megaprosthesis [17], though it is not clear if these results can be expected in case of reconstruction in metastatic lesions. The objective of this systematic review is to collect the available data on elbow metastasis in order to gain a deeper understanding of the primary tumors linked with this condition. Additionally, the aim is to establish whether surgical resection should be followed by reconstructive surgery. Furthermore, the most commonly utilized reconstructive techniques will be outlined, along with their indications and the potential complications associated with these procedures.

## 2. Materials and Methods

### 2.1. Search Strategy

A systematic review of the literature indexed in the PubMed, MEDLINE, and Cochrane Library databases, using as search terms “((metastases) OR (metastasis) AND ((elbow) OR (distal humerus) OR (ulna) OR (radius) OR (radial head) OR (olecranon) OR (upper arm))”, was performed in April 2024. To minimize the number of missed studies, no filters were applied to the search strategy. The bibliography of the selected studies was accurately searched by hand, to identify further studies not found during the electronic search. No restrictions were applied concerning the date of publication. The title of the journal, name of authors, or supporting institutions were not masked at any stage. The Preferred Reporting Items for Systematically Reviews and Meta-analyses (PRISMA) were followed (latest PRISMA statement) [18]. Studies were eligible if they reported at least one patient with metastatic bone disease involving the elbow region (distal humerus, proximal ulna, or proximal radius). For studies reporting multiple skeletal sites, only elbow-specific data were extracted. We excluded studies without extractable elbow-specific data and also recurrences of primary elbow tumors. Furthermore, the management of the metastasis had to be explicitly described. Only full-text articles in English were considered. Review articles and cadaveric and animal studies were excluded.

### 2.2. Study Selection

Two independent reviewers (G.M. and G.G.M.) performed the literature search and reviewed the results. The titles and abstracts were reviewed for all search results, and potentially eligible studies received a full-text review. All differences between the reviewers were discussed, and if disagreement remained, the senior author (R.V.) was consulted.

### 2.3. Data Extraction/Analysis

All the selected studies were retrospectively analyzed by an author (A.D.F.) who then extracted and entered the data in an Excel worksheet. The collected data included the main author, year of publication, article type, number of patients, gender, age, primary tumor, number of metastases and their radiographic characteristics, treatment approach undertaken, and potential complications that occurred. Lastly, the data sheet was reviewed by two authors (M.B.B. and R.V.) who agreed on the extracted data.

### 2.4. Quality Assessment

The methodological quality of the studies was assessed using the mCMS [19]. Each article was evaluated by two independent investigators (A.D.F. and M.B.B.); disagreements >5 points were resolved by a third author. The mCMS (0–100) categorizes studies as excellent (85–100), good (70–84), fair (50–69), or poor (<50). Low mean scores in this review primarily reflect the predominance of case reports/retrospective designs lacking standardized outcomes.

## 3. Results

### 3.1. Search and Literature Selection

**Figure 1:**
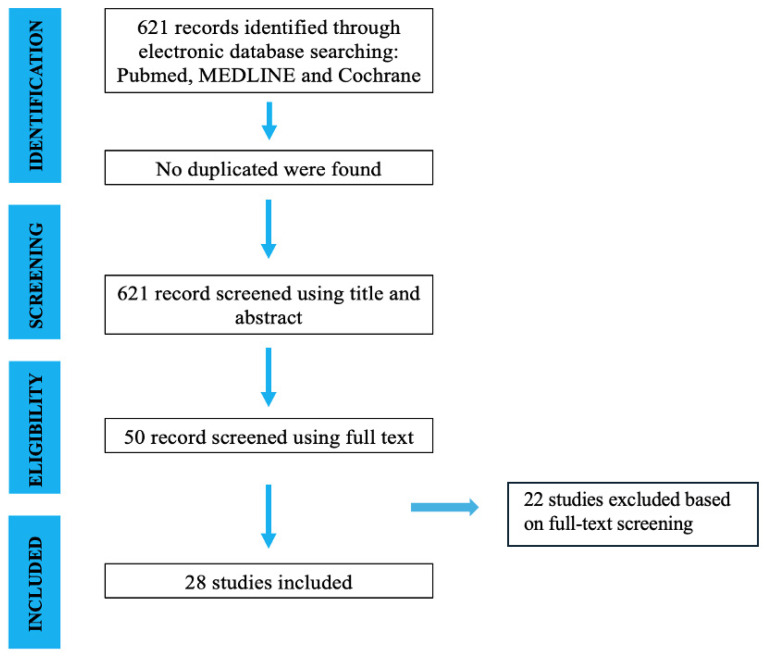
Search and literature selection.

The initial literature search resulted in 621 studies. Once duplicates were removed and the articles were screened for inclusion and exclusion criteria, 50 studies remained, and full texts were assessed for eligibility (Figure 1).

### 3.2. Study Characteristics

A total of 28 articles were included in this systematic review [3,13,18,19,20,21,22,23,24,25,26,27,28,29,30,31,32,33,34,35,36,37,38,39,40,41,42,43]. Of the 28 studies, 19 (68%) were case reports, 7 (25%) were retrospective, 1 (3.5%) was a prospective study, and 1 (3.5%) was a cross-sectional study. Quality scoring through the modified Coleman Methodology Score was then performed, and the mean score of the studies reached was 31 points (10–59 points), which represents a poor study design (Table 1).

### 3.3. Demographic Characteristics

We evaluated 103 patients. Gender was reported for 41 patients (71% male, 29% female), whereas it was not specified for the remaining 62 patients. The mean age at diagnosis was 55 years old (Table 1).

### 3.4. Metastases

We collected information for a total of 103 metastases. As for the primary tumor, renal cell carcinoma (n = 28), breast cancer (n = 9), and lung cancer (n = 6) were the predominant causes of the skeletal metastases (Table 2). For 43 lesions, it was not possible to trace the primary tumor.

Concerning the radiographic aspect, 44 (96%) were osteolytic, 2 (4%) were mixed, and none had an osteoblastic appearance. In 57 cases, this information was not explicit.

In most cases, the distal humerus was the most affected segment (n = 88, 85%), followed by proximal ulna (n = 10, 11%) and radial head (n = 4, 4%). Metastases to the elbow were mentioned generically by one author (Table 2).

### 3.5. Treatment

A total of 10 (10%) metastases were treated with a non-surgical approach, mainly because of the poor general clinical condition of the patients due to the oncological disease. More specifically, nine patients (82%) received radiotherapy and eight patients (73%) received chemotherapy. Six patients (55%) received combined palliative chemotherapy and radiotherapy.

In addition, three (27%) patients received bisphosphonates (zoledronic acid or pamidronate), two (18%) patients received denosumab, and finally, one (9%) patient received immunotherapy.

The surgical approach was chosen for 93 (89%) patients to treat extremely painful lesions resistant to conservative therapies. Among them, 45 (48%) patients were surgically treated for symptomatic lesions with wide resection and different reconstruction techniques, while 48 (52%) patients were treated for either pathological or impending fractures.

Depending especially on the patients’ clinical conditions and life expectancy, the authors chose different surgical techniques. Thirty-eight (41%) patients underwent wide tumor resection and elbow prosthesis placement. Concerning more specifically the type of implants, in 14 (37%) cases, a custom-made prosthesis was used; in 17 (45%), a modular system; in 2 (5%), a standard prosthesis; and finally, in 5 (13%) cases, a standard elbow prosthesis combined with allograft. Four (4%) patients underwent tumor resection followed by reconstructive surgery. Concerning the reconstruction,

- Bone allograft stabilized by a reamed intramedullary nail and a short plate in order to avoid rotations was used after partial distal humerus excision in one patient (25%);
- Non-vascularized autologous fibular graft after proximal ulna excision in one patient (25%);
- Radial neck to humerus trochlea transposition after proximal ulna excision in one patient (25%);
- Bone cement and osteostimulative bone substitute to fill the bone defect after ulnar metastasis resection in one patient (25%).

Wide local excision followed by extracorporeal radiation and reimplantation therapy (ECRT) with plate and screw reconstruction was used in two patients (2%). Finally, a case of free pedicle freezing and autograft–prosthesis reconstruction to treat a proximal radius lesion (1%) was described.

Of the patients who underwent surgical treatment, 48 (52%) patients were treated for either pathological fractures or impending fractures mainly of the distal humerus. Of these, 35 (73%) were treated with plate and cement, 2 (4%) with nailing, 8 (17%) with resection and elbow prosthesis placement (5 standard implants and 3 modular systems), and in 3 (6%) cases, the treatment was not specified (“other”).

Finally, only one (1%) of the patients treated surgically appears to have received neoadjuvant radiotherapy, while thirty (33%) received adjuvant radiotherapy.

## 4. Discussion

Orthopedic surgeons are confronted with a considerable challenge when attempting to diagnose elbow tumors. Over the past few decades, there have been promising developments in the diagnosis, management, and prognosis of patients with bone tumors around the elbow. It is becoming increasingly evident that prompt diagnosis and preoperative planning can have a substantial influence on the treatment and prognosis of these patients.

Although the proximal humerus is a common site for metastasis, the elbow is an uncommon location for both primary and metastatic tumors [20,31,32,46]. The humerus represents the primary site within the upper limb and the second most common site for metastasis in the peripheral skeleton [17,47,48]. De Geyer et al. reported that these humeral metastases occur in the midshaft in 42% to 61% of cases and in the proximal humerus in 32% to 45% of cases [2]. They are secondary to a primary cancer in the breast (17–31%), kidney (13–15%), or lung (11–24%) [38,49,50,51]. Otherwise, it is estimated that the incidence of primary bone lesions and bone metastases in the distal humerus is approximately 1% [40,52]. In light of the limitations of the available data on primary elbow tumors, it is important to note that data on metastatic disease are even rarer. However, the distal humerus appears to be the most affected site. Henrichs et al. reported an incidence of less than 1% [3].

Identifying the primary tumor is essential to ensure the most appropriate management of the patient. In our review, we observed that renal carcinoma is the primary tumor most frequently associated with metastatic lesions around the elbow (28%), followed by breast and lung carcinomas. As stated by the literature, metastatic renal cell carcinoma has been identified as one of the most chemioresistant and radioresistant solid tumors, with an average life expectancy of 12 months [31].

Patients with elbow metastasis typically present with fever, soft tissue edema, a change in the size of the mass, and persistent, non-mechanical rest discomfort. These symptoms are more commonly associated with an aggressive tumor. Diagnostic imaging is a crucial element in the evaluation of patients with musculoskeletal tumors. With regard to the radiographic aspect, the majority of secondary injuries localized to the humerus and more specifically to the elbow are osteolytic [2,20,46]. These kinds of lesions result in structural disruption and weakening of the bone architecture, leading to an increased risk of fracture. Our data also confirmed this. In fact, 96% were osteolytic metastases. These conditions frequently manifest as severe pain, which can result in complete loss of upper limb function [2,41].

As “around the elbow” metastases are quite rare, a delay in diagnosis is common. However, it remains crucial to be able to make an early diagnosis as this can have a significant impact on the patient’s prognosis [46]. Precisely because of the uniqueness of this condition, no standard exists to date concerning the management and treatment of these lesions. As a result, it is currently challenging to provide precise guidelines to support surgeons in making the right decisions.

According to Weber KL et al., palliative radiation or limited surgery are the most common treatments in patients with elbow metastasis [10]. However, our data showed that only 11% of the metastases have been treated with a non-surgical approach. In all cases, the conservative approach was chosen, mainly because of the poor general clinical condition of the patients due to the primary tumor. Among them, almost all patients underwent radiotherapy and palliative chemotherapy [10,24,30]. Considering the literature, radiotherapy (RT) is an effective treatment for pain management in patients with bone metastases. [53,54]. Kord et al. treated a bone metastasis in the elbow from uterine carcinosarcoma with denosumab, RT, and CHT [28]. Croucher et al. reported that bisphosphonates inhibit osteoclastogenesis and osteoclast-mediated bone resorption, which may result in indirect anti-tumor effects [55]. Furthermore, according to some authors, bisphosphonates seem to improve the clinical results obtained with radiotherapy [56].

In our review, we found that three patients received bisphosphonates (zoledronic acid or pamidronate) in addition to RT and CHT [33,42,45].

The surgical treatment of metastases around the elbow is definitely more challenging than in other anatomic areas due to the limited soft tissue envelope and the neurovascular structures in close proximity to the tumor. Based on our review, 90% of patients were treated surgically for either a symptomatic lesion or an impending or pathological fracture around the elbow. In our opinion, this high percentage is attributable to the fact that patients with a poor clinical condition and with multiple metastases, both bone and visceral lesion, are frequently not eligible for treatment and represent the majority of the total population of patients. Moreover, the scientific literature focuses more on reporting the surgical cases and it does not provide sufficient data on the real epidemiology of the metastatic lesions of the elbow, including both surgical and non-surgical cases.

Amongst patients treated surgically, 45 (48%) patients were treated for symptomatic lesions with wide resection and different reconstruction techniques, while 48 (52%) patients were treated for either pathological or impending fractures with operative fixation or elbow replacement. In both cases, it requires careful preoperative planning and consideration of several possible resection and reconstruction techniques according to the size and location of the tumor. The surgical treatment options may include osteoarticular allografts, APC (allograft–prosthesis composite), custom-made and modular prosthesis, plate and cement fixation, and nailing. Therefore, when possible, a more radical and definitive treatment is suggested [28]. We noted that none of the authors choose arthrodesis as a surgical treatment. According to the literature, this technique has poor tolerability by patients, and it is associated with severe functional limitation [57,58]. In addition, although limb amputation was the treatment of choice in the past, it was not mentioned by any author [48]. This is thanks to the combination of adjuvant chemotherapeutic protocols and improved radiation therapy techniques in association with ‘en bloc’ resection of the tumor and various limb salvage and reconstruction procedures [59].

Of the 45 patients treated for symptomatic lesions after failure of conservative therapies, 38 patients underwent wide tumor resection and elbow prosthesis placement [7,12,15,21,31,35,37]. With regard to the type of implant used, it seems that the various authors preferred to use modular prostheses rather than custom-made ones [7,12,15,21,31,35,37]. Only two patients had a conventional prosthesis implanted [15]. Based on the literature, it seems better to use modular prostheses or mega prostheses than conventional ones to manage the bone defect around the elbow [45,57,58]. The advantages of prosthetic elbow replacement are the restoration of bone defects with satisfactory function and pain relief, and a relatively low number of complications compared to other techniques such as allografts or arthrodesis [24,28,49,53,54]. The most common complications related with these techniques are poor wound healing, infection, radial and ulnar nerve palsy, and mechanical complications [17,45,57,58]. Casadei et al., out of nine cases, had one case of ulnar palsy, one aseptic loosing, and two local recurrence [31]. It is notable that the complication rate in this study was not found to be related to radiotherapy, which differs from the findings of Ratasvuori, who observed that cases with complications were more likely to have undergone radiation therapy than those without complications, regardless of the surgical reconstruction technique employed [7].

In another study, he reported only one case of infection out of seventeen patients operated on, which, however, necessitated revision of the implant [15]. Sorensen et al., in their study, reported two complications out of five procedures. One was mechanical and the other was an infection [37]. Instead, Liao et al. reported no complications after the use of the custom-made prosthesis based on 3D printing technology [21]. He also reported good functional outcomes based on the Mayo Elbow Performance Score (MEPS).

In five cases, a standard elbow prosthesis combined with allograft (APC) was used with fair functional outcomes compared with modular prosthesis [15]. In fact, the MEPS score was lower if compared with that of patients undergoing prosthesis replacement [12] According to the literature, the use of APC reduces the risk of joint instability, but it is still associated with high complication rates such as deep infections and allograft fractures [12].

Only 4 out 45 patients treated for symptomatic lesions underwent tumor resection followed by reconstructive surgery [14,44]. All authors chose a different reconstructive technique, emphasizing that the choice is related to both surgeon preference and characteristics, as well as lesion size. Fernández-Valencia JA et al. decided to use an osteoarticular allograft since they had experience and good results in using this procedure [43]. The patient suffered a radial nerve paralysis, which was resolved with the aid of rehabilitation and non-union at the graft–host junction, which responded to autogenous bone grafting [43]. Aljuhani WS et al. used radial neck-to-humerus trochlea transposition after proximal ulna excision because the patient rejected an above-elbow amputation: so, they considered the reconstructive option with the least possible side effects and probability of revision surgery [21]. In the literature, we found that the complication rate related to this surgery is between 33% and 70%, and the most common complications are infection and instability of the joint [58,59,60]

Regarding the patients who underwent surgical treatment, 48 (52%) were either treated for pathological fractures or impending fractures mainly located at the distal humerus. According to the literature, patients often undergo surgery for impending or pathological fractures with the goal of function restoration and pain reduction [47]. While dual plate and screw fixation (PSF) is widely regarded as the gold standard for treating distal humerus fractures caused by trauma, there is no clear consensus on the best operative approach for managing distal humeral pathological or impending pathological fractures [20]. This uncertainty likely stems from the wide range of underlying conditions, unique patient factors, and limited literature about this topic. There are several treatment options, including conservative management, intramedullary nailing, endoprosthetic elbow replacement, and PSF with either one or two plates [60,61,62,63,64]. According to the literature, PSF with one plate is the most common procedure [38,64]. Although, two studies in the literature report that treating such lesions with a single plate is associated with a high revision rate (30%), often requiring the addition of a second plate to improve the stability [31,38]. Another study found that dual plate and screw fixation (PSF) could withstand significantly higher peak torque and absorb more energy compared to single plate fixation, suggesting that dual PSF may be a more effective first-line treatment rather than a salvage option [65].

In our review, the most common surgical procedures for patients affected by impending or pathological fracture were plate and cement (73%), nailing (4%), resection and elbow prosthesis placement (17%), while in 6% cases, the treatment was not specified. According to the criteria of Capanna and Campanacci, resection with arthroplasty reconstruction should be performed in case of pathological fractures or in case of lesions with a high risk of fracture if the patient’s survival is expected to be more than 6–12 months [55]. In most of the article, the use of one or two plates was not specified. Wedin R. et al. reported 15 cases of pathological fracture of the distal humerus treated mostly with plating [15]. Instead, West W. et al. reported 35 cases, where 21 underwent single plating and 14 double plating [18]. According to the literature, the incidence of complications ranges from 30% to 50%, and the most common are superficial and deep infection, non-union, deep vein thrombosis, tumor progression, and radial nerve palsy [39,48]. Wedin R. et al. found a complication rate of 33%, but in half of the cases, they resolved spontaneously [38], while West W. et al. reported a complication rate of 23%. They found more complications in patients treated with single plating (28.6%) than in those treated with double plating (14.3%), but this was not a significant difference [20].

According to the data we collected for this systematic review, a surgical indication consists both of extremely painful lesions resistant to conservative therapies and patients with pathological fractures. When the surgeon faces a condition such as distal humerus pathological fracture or impending fracture, single or double plate fixation represents the best treatment option [37]. Double plate fixation is not inferior to single plate fixation and potentially results in fewer reoperations and better functional outcomes in the postoperative period [19], though this technique may require a longer surgery time, which is associated with higher blood loss and infection risks [47]. In addition, the use of another plate on the medial side can be associated with ulnar lesions. Instead, in cases where patients have a limited life expectancy, single plating may be favored to reduce immediate surgical risks, as long-term stability is not essential [20].

For patients with painful lesions that are resistant to conservative therapies, the most common treatment is bone tumor resection followed by prosthetic replacement. In these cases, modular prostheses or megaprostheses are generally preferred over standard prostheses due to their ability to better accommodate bone defects and provide improved long-term function. These prosthetic options offer greater adaptability and are associated with better clinical outcomes, especially in complex cases [45,57,58].

The present study has several limitations. First, due to the rarity of elbow metastases, the study population was relatively small. Second, because of the types of articles available, most of the included studies were case reports or small case series. Since the scientific literature does not provide clinical studies with large patient cohorts, we also included these low-level designs, which may introduce bias. Furthermore, demographic and clinical data were frequently incomplete: in 62 out of 103 patients, gender was not specified, and in almost 40% of cases, the primary tumor was not reported. In addition, none of the included studies systematically described treatment-related complications or reported standardized clinical or functional outcome scores. Another limitation is the potential overlap of patient cohorts among studies from the same institutions, which could not be fully excluded. For this reason, the conclusions deduced within this study are limited a low level of evidence from the included studies.

## 5. Conclusions

Elbow metastasis is a rare condition, and due to its infrequency, the scientific literature lacks precise guidelines to guide surgeons in its management. When treating patients with elbow metastasis, a thorough evaluation is crucial, considering the patient’s functional status, pain management needs, and overall prognosis, which influence the choice of treatment. The selected treatment should aim to provide optimal functional outcomes and minimize complications. For patients with pathological or impending fractures, single or double plate fixation is typically the preferred approach. In cases where patients have a limited life expectancy, single plating may be favored to reduce immediate surgical risks, as long-term stability is not essential. For patients with severe, symptomatic lesions unresponsive to conservative therapy, resection followed by the implantation of a modular prosthesis usually offers the best clinical and functional outcomes.

## Figures and Tables

**Table 1:** Studies and demographic characteristics. mCMS: Modified Coleman Score; x: data not available.

Authors	Year of Publication	Type of Article	mCMS	n° Patients	Gender		Age at Diagnosis (Years)
					Male	Female	
Henrichs MP et al. [3]	2018	Retrospective Study	55	2	x	x	69
Casadei R et al. [15]	2018	Retrospective Study	45	9	7	2	64
West W 3rd et al. [20]	2024	Retrospective Study	35	23	x	x	64
Liao GJ et al. [21]	2023	Case Report	32	1	1	-	56
Scherer S et al. [22]	2023	Case Report	30	1	1	-	6
Aljuhani WS et al. [23]	2023	Case Report	27	1	1	-	60
Gamage S et al. [24]	2023	Case Report	12	1	-	1	68
Boddu D et al. [25]	2021	Case Report	37	1	1	-	7
Luo Z et al. [26]	2021	Case Report	17	1	1	-	68
Bianchi A et al. [27]	2020	Case Report	22	1	1	-	80
Kord A et al. [28]	2020	Case Report	10	1	-	1	52
Vempuluru VS et al. [29]	2020	Case Report	25	1	1	-	0.9
Cai L et al. [30]	2020	Case Report	22	1	1	-	62
Casadei R et al. [31]	2016	Retrospective Study	52	17	x	x	58.5
Megas P et al. [32]	2017	Case Report	18	1	1	-	50
Devdas SK et al. [33]	2016	Case Report	20	1	-	1	52
Subhadrabandhu S et al. [34]	2015	Retrospective Study	55	1	1	-	65
Pruksakorn D et al. [35]	2014	Prospective Cohort Study	59	3	1	2	X
Tunio MA et al. [36]	2013	Case Report	22	1	-	1	73
Sørensen MS et al. [37]	2013	Cross-Sectional Study	43	5	x	x	64
Wedin R et al. [38]	2011	Retrospective Study	56	15	x	x	67
Culleton S et al. [39]	2008	Case Report	23	1	1	-	51
Hanna SA et al. [40]	2007	Retrospective Study	54	6	4	2	60
Rolf et al. [41]	2004	Case Report	37	4	4	-	65.2
Ansari et al. [42]	2003	Case Report	13	1	1	-	60
Fernández-Valencia JA et al. [43]	2002	Case Report	33	1	-	1	48
Durusu et al. [44]	2002	Case Report	13	1	-	1	38
Peterson JJ et al. [45]	1999	Case Report	13	1	1	-	79
			31	103	29	12	55

**Table 2:** Metastases’ characteristics; x: data not available.

Authors	n° Metastasis	Localization				Primary Tumor			
		Distal Humerus	Proximal Radius	Proximal Ulna	“Elbow”	Breast	Lung	Kidney	Other
Henrichs MP et al. [3]	2	2	-	-	-	-	-	2	-
Casadei R et al. [13]	9	9	-	-	-	-	-	9	-
West W 3rd et al. [18]	23	23	-	-	-	x	x	x	x
Liao GJ et al. [19]	1	1	-	-	-	-	-	1	-
Scherer S et al. [20]	1	-	-	1	-	-	-	-	Neuroblastoma (1)
Aljuhani WS et al. [21]	1	-	-	1	-	-	-	-	Colon (1)
Gamage S et al. [22]	1	-	1	-	-	-	1	-	-
Boddu D et al. [23]	1	-	-	1	-	-	-	-	Retinoblastoma (1)
Luo Z et al. [24]	1	-	-	-	1	-	1	-	-
Bianchi A et al. [25]	1	1	-	-	-	-	-	-	Colon (1)
Kord A et al. [26]	1	-	-	1	-	-	-	-	Uterus (1)
Vempuluru VS et al. [27]	1	-	-	1	-	-	-	-	Retinoblastoma (1)
Cai L et al. [28]	1	-	-	1	-	-	1	-	-
Casadei R et al. [29]	17	17	-	-	-	5	2	6	Osteosarcoma (1), Bladder (1), Liver (1), Thyroid (1)
Megas P et al. [30]	1	-	-	1	-	-	-	1	-
Devdas SK et al. [31]	1	1	-	-	-	-	-	-	Uterus (1)
Subhadrabandhu S et al. [32]	1	-	1	-	-	-	-	1	-
Pruksakorn D et al. [33]	3	2	-	1	-	2	1	-	-
Tunio MA et al. [34]	1	1	-	-	-	-	-	-	Gallbladder (1)
Sørensen MS et al. [35]	5	5	-	-	-	x	x	x	x
Wedin R et al. [36]	15	15	-	-	-	x	x	x	x
Culleton S et al. [37]	1	-	-	1	-	-	-	1	-
Hanna SA et al. [38]	6	6	-	-	-	1	-	3	Melanoma (1), Larynx (1)
Rolf et al. [39]	4	3	-	1	-	-	-	4	-
Ansari et al. [40]	1	-	1	-	-	-	-	-	Prostate (1)
Fernández-Valencia JA et al. [41]	1	1	-	-	-	1	-	-	-
Durusu et al. [42]	1	-	1	-	-	-	-	-	Neuroblastoma (1)
Peterson JJ et al. [43]	1	1	-	-	-	-	-	-	Squamous Skin (1)
	103	88	4	10	1	9	6	28	17

## Data Availability

No new data were created or analyzed in this study. Data sharing is not applicable to this article.
